# A Mood Semantic Awareness Model for Emotional Interactive Robots

**DOI:** 10.3390/s24030845

**Published:** 2024-01-28

**Authors:** Tiehua Zhou, Zihan Yu, Ling Wang, Keun Ho Ryu

**Affiliations:** 1 Department of Computer Science and Technology, School of Computer Science, Northeast Electric Power University, Jilin 132013, China; thzhou@neepu.edu.cn (T.Z.); 2202100988@neepu.edu.cn (Z.Y.); 2 Department of Computer Science, College of Electrical and Computer Engineering, Chungbuk National University, Cheongju 28644, Republic of Korea; khryu@chungbuk.ac.kr; 3 Data Science Laboratory, Faculty of Information Technology, Ton Duc Thang University, Ho Chi Minh City 700000, Vietnam; 4 Biomedical Engineering Institute, Chiang Mai University, Chiang Mai 50200, Thailand

**Keywords:** dialogue modeling, emotional interactive robots, continuation of the topic, semantic interaction service

## Abstract

The rapid development of natural language processing technology and improvements in computer performance in recent years have resulted in the wide-scale development and adoption of human–machine dialogue systems. In this study, the Icc_dialogue model is proposed to enhance the semantic awareness of moods for emotional interactive robots. Equipped with a voice interaction module, emotion calculation is conducted based on model responses, and rules for calculating users’ degree of interest are formulated. By evaluating the degree of interest, the system can determine whether it should transition to a new topic to maintain the user’s interest. This model can also address issues such as overly purposeful responses and rigid emotional expressions in generated replies. Simultaneously, this study explores topic continuation after answering a question, the construction of dialogue rounds, keyword counting, and the creation of a target text similarity matrix for each text in the dialogue dataset. The matrix is normalized, weights are assigned, and the final text score is calculated. In the text with the highest score, the content of dialogue continuation is determined by calculating a subsequent sentence with the highest similarity. This resolves the issue in which the conversational bot fails to continue dialogue on a topic after answering a question, instead waiting for the user to voluntarily provide more information, resulting in topic interruption. As described in the experimental section, both automatic and manual evaluations were conducted to validate the significant improvement in the mood semantic awareness model’s performance in terms of dialogue quality and user experience.

## 1. Introduction

Research on human–computer communication with emotion aims to study the communication between a system and its user. Studies have shown that emotions can cause specific reactions and affect decision making [1]. Therefore, communication modes with emotional semantic perception can improve the human–computer interaction experience. The objective of studying emotional communication in human–machine interactions is to equip machines with the capability to detect and convey emotions. This, in turn, enables machines to engage with users in a human-like way—a fundamental aspiration in the ongoing evolution of artificial intelligence. Semantic interaction services with voice functions can provide feedback on various tasks with a sense of emotion, which is a core technology in improving user experience [2].

In recent years, preliminary research results have been obtained on the use of intelligent chatbots, including Apple’s Siri, Baidu’s Xiaodu, Huawei’s Xiaoyi, and so on. These chatbots’ main function is to carry out a user’s simple instructions, such as playing music, checking the weather, viewing the time, etc., mainly consisting of task-based dialogue. In both task-oriented and open-domain scenarios within contemporary human–computer interaction research, most methodologies define a conversation as a task that produces an output response based on user input and the contextual dynamics of the ongoing dialogue. The majority of response-generation models adhere to text-generation frameworks like sequence-to-sequence (Seq2Seq), conditional variational autoencoders (CVAE), and generative adversarial networks (GANs). These models are trained on extensive conversational datasets, enabling them to provide contextually relevant responses across a broad spectrum of user interests. However, the replies generated using these frameworks have a strong purpose, stiff emotions, and less-than-ideal anthropomorphism, and ignore the emotion being expressed by the user statement, which is a major problem in open-domain dialogue. Therefore, the incorporation of emotional elements into the realm of human–computer interaction is now becoming a pivotal focus within the research and development domain of intelligent robots.

Based on the above problems, this paper proposes a human–computer interaction method based on emotional communication that combines a large number of Twitter data and an emotion dictionary to generate a word frequency table of interest, formulate a calculation rule to determine interest level, determine whether to switch topics according to the user’s interest level, and locate the index text to respond to. In order to make the method more anthropomorphic and enable continuous dialogue, the dialogue will continue after answering the question. The approach used in this study is to construct a feature matrix and calculate the interest score of the user based on the user statement and text dataset in order to locate the most suitable response text for dialogue continuation. Using the method proposed in this article, stilted chatbot conversations can take on an emotional element, and topics that the user is more interested in can be found to allow the conversation to continue.

## 2. Related Work

In the past few years, the rapid advancements in natural language processing technology and significant improvements in computer performance have allowed human–machine dialogue systems to be widely developed and applied. According to their application scenarios, interactive systems with artificial intelligence capabilities can be categorized into two main types: open-domain and closed-domain human–computer dialogue systems [3,4]. This section will focus on the research status of human–machine dialogue systems in both of these application fields.

Open-domain human–machine dialogue systems aim to produce coherent responses to users through multiple rounds of dialogue [5]. The complexity in this domain lies in incorporating both syntactic and semantic aspects of language when generating responses. Although early dialogue systems made some progress in specific environments, they had limited effectiveness in a wide range of scenarios. In recent times, there has been a concerted effort to enhance the anthropomorphic characteristics of robots, and researchers have focused on open-domain human–machine dialogue systems, resulting in the emergence of many valuable human–machine dialogue models [6,7].

Zhou et al. proposed that the Emotion Chat Machine (ECM), through an innovative mechanism, can not only achieve accurate responses to content in large-scale dialogue generation but can also effectively demonstrate emotional consistency in their responses. Their study was the first attempt to address emotional problems in this area [8]. Ling et al. introduced a model known as context-enhanced neural question generation (CNQG), designed to enhance the interactivity and continuity of multi-round conversations by generating questions in multiple rounds of conversation using encoder–decoder frameworks and attention mechanisms. The experimental results showed that CNQG outperformed the baseline model in a number of evaluation indicators, solved the problem of response generation in multiple rounds of open-domain dialogue for the first time, and was able to proficiently capture features from larger contexts while retaining efficacy in specific contexts [9]. Wang et al. proposed two novel models, SentiGAN and C-SentiGAN, which could automatically generate text with different emotional labels through multiple generators and multi-class discriminators. These models adopted penalty-based goals to generate diversified samples of specific emotional labels and ensure quality. The experiments showed that SentiGAN is superior to other models in terms of the emotional accuracy and quality of the generated text, and C-SentiGAN is outstanding in conditional text-generation tasks [10].

Closed-domain human–machine dialogue systems are usually targeted for specific tasks, have clear goals and limited knowledge scopes, and are regarded as helpers in specific fields, dedicated to providing services to users [4]. Because these systems are applied in specific scenarios, the fault tolerance is low, but compared with open-domain dialogue systems, closed-domain systems can meet the highly specific needs of their users. The closed-domain system depends on its external knowledge base being supplemented and updated for optimal functioning. In addition, the context of user input is more logical and coherent, and there is a strong correlation between emotions [11,12].

The traditional recommendation system is static and cannot dynamically adjust its recommendations according to the user’s requirements and emotions. Kang D. and others proposed an interactive dialogue recommendation approach, in which recommendations are taken as dialogue tasks, allowing experts to gradually learn about a user’s needs and recommend them products. A goal-driven recommendation dialogue dataset was created, and a dialogue recommendation system was developed. Through pre-training and fine-tuning, the system was able to learn better strategies for cooperative dialogue. The experiments showed that the pre-trained, fine-tuned system outperforms non-fine-tuned models in achieving dialogue goals, and it was also evaluated more consistently by users [13]. Task-based dialogue systems can also be applied to performing tasks in specific fields, such as product recommendations, restaurant reservations, and even price discussions through the dialogue system [14,15,16,17].

In the emotional interaction between a human and a machine, the agent plays a key role. In a specific interactive task, emotion is embodied as emotion, and the agent’s autonomous perception and emotional expression are important characteristics through which the agent can perceive users’ emotions and states, generate appropriate emotional responses through internal learning adjustments, promote emotional communication, and even affect users’ emotions [18,19]. Quan et al. used the combined cepstrum distance method to identify emotion in speech [20]. Rao et al. reviewed sentiment computing technologies based on semantic analysis [21]. Kanjo et al. employed deep learning to classify emotions by iteratively adding and removing large numbers of sensor signals from different modes. Their dataset was sourced from smartphones and wearable devices in the real world, and integrated three sensor modes, namely body, environment, and location, to build a global model reflecting the temporal relationship between signal dynamics and modes [22]. Ma et al. proposed an approach called the depth-weighted fusion method for the recognition of emotions in audiovisual content. In their approach, by employing cross-modal noise modeling, both audio and video data undergo a purification process, voice activity detection is performed using VAD, and redundancy within visual data is addressed by aligning speech regions. A CNN is utilized to extract audio and visual features, followed by the integration of multimodal features through deep belief networks. Transfer learning was used to train the network, and emotion classification was carried out in combination with support vector machines, taking into account factors such as cross-modal feature fusion, denoising, and redundancy [23]. Chen et al. adopted support vector machine and random forest methods as emotion classifiers to select the best hyperparameters through a grid search to obtain an optimized model [24]. At the same time, in order to carry out emotional recognition in a multicultural environment, they introduced the hidden-layer features of emotional correlation and cultural correlation.

Table 1 summarizes the research status of evaluation tools, open-domain human–computer dialogue systems, and closed-domain human–computer dialogue systems. A synthesis of the above literature shows that open-domain human–computer dialogue systems are suitable for multidomain and multitopic dialogues and are able to deal with a wide range of questions and topics raised by users, but due to their broad nature, they can suffer from problems such as a general lack of information. Closed-domain human–computer dialogue systems target specific domains or tasks in a limited number of domains, and while this makes these systems more likely to understand the user’s intentions within these domains, they cannot handle a wide range of topics beyond their original scope. In response to this issue, we propose the model in this paper, which integrates the advantages and disadvantages of the open-domain and closed-domain systems mentioned above, calling on the open-domain model when answering a question and calling on inverted indexing when continuing the dialogue. Our model takes into account both the relevance of answering a question and the problem of information scarcity. For comprehensive evaluation of our model, BLEU [25] automatic evaluation and manual evaluation were chosen. Finally, since we integrate emotional factors in this study, we measure emotional consistency and average response length, as these metrics are more suitable for this type of model measurement.

## 3. Materials and Methods

### 3.1. Motivation

With the continuous development of dialogue systems, there is an increasingly clear trend of strengthening the integration of emotional factors within these systems. Although traditional dialogue systems can handle basic verbal communication, their responses are often too mechanical, lacking in emotional resonance. In order to get closer to the essence of communication, dialogue systems urgently need to introduce richer emotional factors. Emotion is not only a key element of interaction, but is also the soul of communication, adding depth, authenticity, and resonance to a conversation. Therefore, by incorporating emotional elements into the conversation system, we can improve its interactivity, increase user satisfaction, and create a more personalized conversation experience.

**Figure 1:**
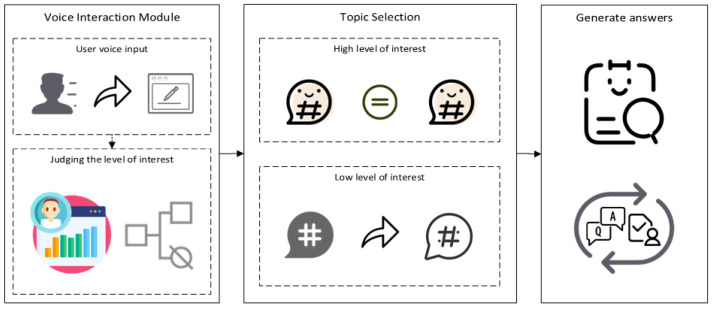
Mood semantic awareness model flowchart.

Therefore, we carried out emotion calculation based on the model’s answers and formulated the calculation rules of user’s degree of interest. According to the degree of interest results, the model can determine whether to switch topics, so as to maintain the user’s high degree of interest in the conversation. Additionally, existing models also have a tendency to interrupt the conversation after answering the current question. This means that it may answer a user’s question without moving the conversation along, as it is waiting for the user to volunteer more information or questions. This may affect the continuity of the dialogue and reduce the desire of users to continue chatting. Therefore, this model continues the dialogue after the user’s question is answered; constructs rounds of dialogue based on each text in the dialogue dataset, the number of keywords, and the similarity matrix of the target text; and then normalizes the matrix, assigns weights, and computes the ultimate textual score. The text content of the next sentence is then determined based on the most similar sentence to continue the conversation. A flowchart of the mood semantic awareness model is shown in Figure 1 below.

### 3.2. Dataset

In terms of word frequency table statistics, the Sentiment140 dataset [26] is widely used in sentiment analysis tasks. The dataset, created by researchers at Stanford University, contains about 1.6 million messages from Twitter that are classified as containing either positive and negative emotions, which are used to train and evaluate sentiment analysis models.

For question-answering processing, we used the CMU_DoG [27] dataset, which is a document-based text conversation dataset. The term “document-based conversation” here refers to conversations that relate to the content of specific documents. The CMU_DoG dataset comprises designated documents, specifically Wikipedia articles discussing popular movies. It encompasses a total of 4112 conversations, averaging 21.43 sessions per conversation. This means that this dataset can not only draw on relevant chat history when generating responses, but can also serve as a source of information for our model.

By combining these two datasets, we can perform sentiment analysis tasks and document-based conversation generation tasks, respectively. This allows us to calculate emotional word frequency tables from the Twitter dataset, develop rules for calculating interest levels, and train the model to generate coherent, relevant responses when generating conversation.

### 3.3. Moods Semantic Awareness Model

The whole process of the Icc_dialogue model consists of the following main modules: The first is a text information collection module, which is used to collect, classify, and preprocess text data, in addition to obtaining social data, sorting and cleaning original text, and building social text datasets. Next, based on the social text dataset and emotion dictionary [28], the interest index guidance module extracts sentences containing keywords relating to interest level, calculates the occurrence frequency of various emotion words in the sentences according to 22 categories of emotion in the emotion word dictionary, generates emotion word frequency tables, and calculates the corresponding degree of interest of different emotion words. According to the degree of interest and the length of the dialogue statement, the degree of interest of the dialogue statement is calculated. The dialogue interaction module organizes and cleans the dialogue corpus, generates the dialogue dataset, reads the user input text, establishes a two-level index, and locates the relevant text from the dialogue dataset according to the keywords contained in the question. After text positioning, the model answer is used to output the answer to the question. The topic continuity in the dialogue is judged based on the calculation of the degree of interest, and the range of the primary index is determined. In the dialogue continuation module, in the content of the secondary index, the rounds of dialogue, keywords, and target text similarity matrix of each text in the dialogue dataset are constructed, the matrix is normalized, weights are assigned, and the final text score is calculated. The text content of the next sentence is calculated based on the highest similarity with the text with the highest score and then used to continue the dialogue. These modules together form the complete mood semantic awareness model.

## 4. Emotion Intensity Division and Calculation of Interest

**Figure 2:**
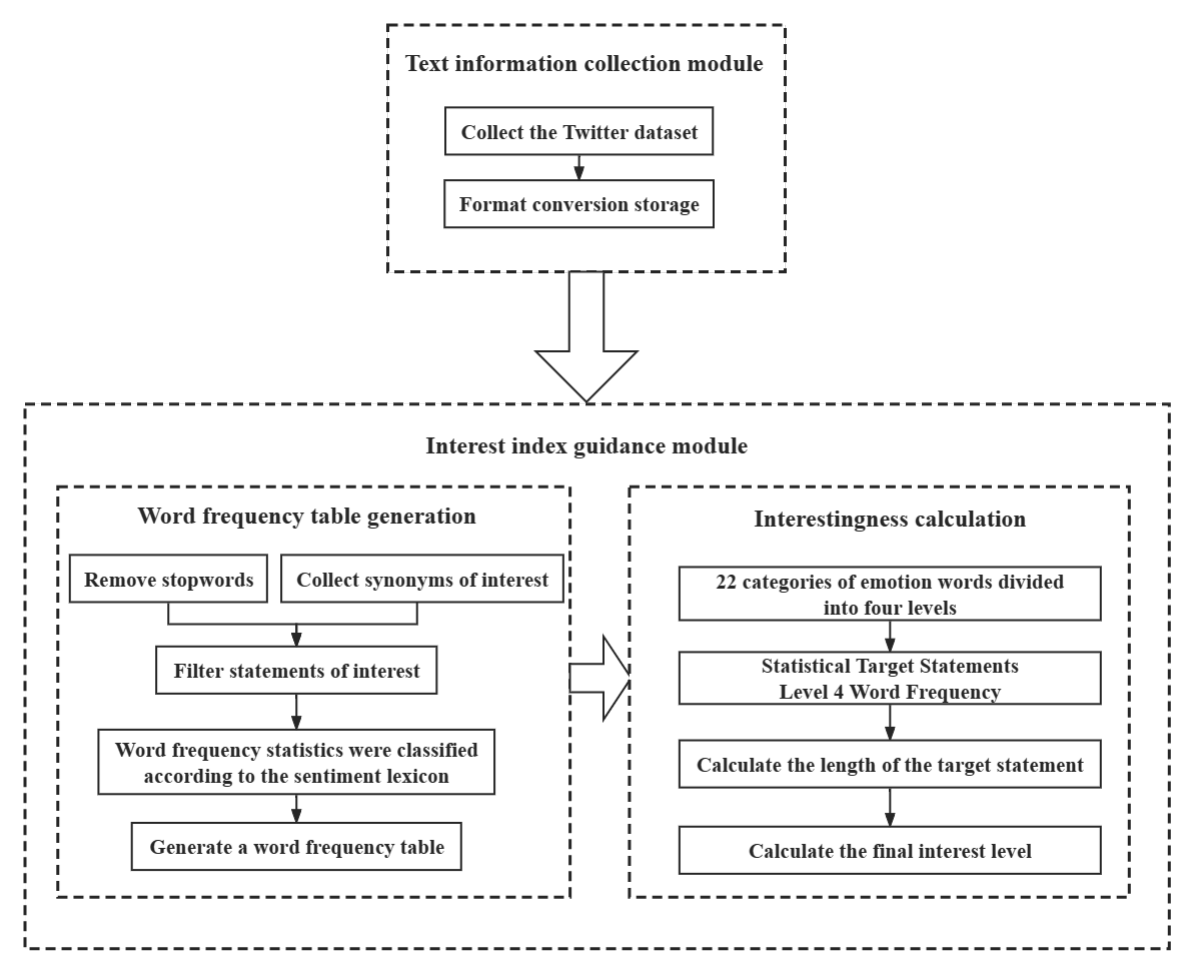
Division of emotional intensity and calculation of interest flowchart.

In this section, large-scale social text data are processed and analyzed: the number of emotional words is counted, the emotional word frequency table is generated, and the emotional word categories are divided into boxes according to set rules to classify different emotional intensities. Based on the division of emotional words by intensity and the sentence length statistics, the calculation rules for the degree of interest are formed, which are used to calculate the interestingness of the topic in the subsequent dialogue to determine whether to continue with the current topic or switch topics. The flowchart of this section is shown in Figure 2.

### 4.1. Division of Emotional Intensity

In this section, our first task is to divide the intensity of the 22 types of emotions in the existing sentiment dictionary. The 22 emotional words we use are as follows: Concentration, Rage, Depression, Joy, Despair, Stress, Pain, Respect, Surprise, Passion, Fear, Anger, Gratitude, Hope, Disgust, Confidence, Sorrow, Intimacy, Shame, Boredom, Insecurity, and Manic. These emotions were divided into four levels for the subsequent calculation of interest. In this section, we determine sentiment intensity partitioning rules by processing large-scale social text data, analyzing a total of 1.6 million tweets.

In order to maintain the data’s quality and uniformity, we preprocess the raw Twitter data. First, we remove duplicate tweets and ensure that each tweet is unique to avoid the impact of duplicate data on subsequent analysis. In addition, since our study focuses on English texts, we removed non-English tweets to ensure data consistency. Finally, we normalized the format of the tweets’ text. We removed special characters, URLs, and punctuation in the tweet text to ensure cleanliness. In this way, noise and interference can be reduced, making subsequent text analysis more accurate.

**Figure 3:**
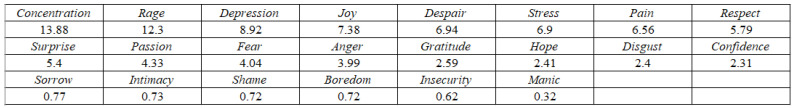
Word frequency statistics table.

Words indicating interest, such as interesting, fascinating, absorbing, and other synonyms, including synonyms and words in different tenses, were collected to form a dataset of words of interest $W=w_{1},w_{2},\ldots ,w_{i},\ldots ,w_{n}$, where $w_{i}$ is the *i*th word of interest. Sentences from the processed Twitter text data containing words in the set of words of interest *W* were extracted to form the set $S=s_{1},s_{2},\ldots ,s_{i},\ldots ,s_{n}$, where $s_{i}$ is the *i*th statement in the set containing keywords of interest. Word segmentation processing was then performed on the sentences in the set *S*, and word frequency statistics were performed based on 22 words from emotional dictionaries, sorted by word frequency; the generated word frequency statistics table is shown in Figure 3.

According to the word frequency statistics of this table, the 22 kinds of emotion words were divided into four kinds of emotional intensity: HH,H,M,L. HH represents a high degree of interest, *H* represents a medium degree of interest, *M* represents a general degree of interest, and *L* represents a low degree of interest. HH includes three emotional words: Concentration, Rage, and Depression. *H* includes nine emotional words: Joy, Despair, Stress, Pain, Respect, Surprise, Passion, Fear, and Anger. *M* includes four emotional words: Gratitude, Hope, Disgust, and Confidence. Finally, *L* includes six emotional words: Sorrow, Intimacy, Shame, Boredom, Insecurity, and Manic.

### 4.2. Calculation of Interest

In the calculation of interest, firstly, according to the division of emotional intensity outlined in Section 4.1, the user input sentence is analyzed and categorized based on the number of emotional words it contains, and the level of interest is sorted according to the number interest levels, where the first two degrees of interest are used to calculate and determine the interestingness of the topic. The degree of interest classification includes Interest={HH,H,M,L}, and the first two degrees of interests are $Interest_{Top_{1}}$ and $Interest_{Top_{2}}$. The number of emotion words corresponding to the *i*th degree of interest in the sentence is $N_{Interest_{Top_{i}}}$, and the ratio of the number of emotion words corresponding to each degree of interest in the sentence to the number corresponding to the first two degrees of interest $Rate_{Interest_{Top_{i}}}$ is calculated as follows:

(1) $$Rate_{Interest_{Top_{i}}}=\frac{N_{Interest_{Top_{i}}}}{\sum_{i=1}^{2}N_{Interest_{Top_{i}}}}$$

Here, the topic’s degree of interest is set as $Interest_{Topic}$, and the calculation rules for the topic’s degree of interest are shown in Table 2. In this table, 75%, 65%, 50%, 25% are the test values used in the test process, as statistical analysis shows that when these values are taken, an overall balance can be achieved.

Observing a large dialogue corpus, it is found that there is a certain correlation between the degree of interest of the topic and the length of the sentence. Therefore, the algorithm adds a sentence length limit while calculating the sentiment of words. Length statistics are carried out on the large-scale dialogue datasets to determine the text length threshold, where the 30% threshold is taken as the low threshold $num_{s}$ and the 70% threshold is taken as the high threshold $num_{b}$. Moreover, num represents the length of the sentence in the user input text, where the unit is words. If $num\leq num_{s}$, the level of interest is reduced by one level, that is, from HH to *H*, *H* to *M*, *M* to *L*, and *L* remains the same. If $num\geq num_{b}$, the level of interest is increased by one, that is, from *L* to *M*, *M* to *H*, *H* to HH, and HH remains the same, and the result is used as the final $Interest_{Topic}$. In this section we developed calculation rules to calculate and analyse the level of interest.

## 5. Sustainable Dialogue Systems

**Figure 4:**
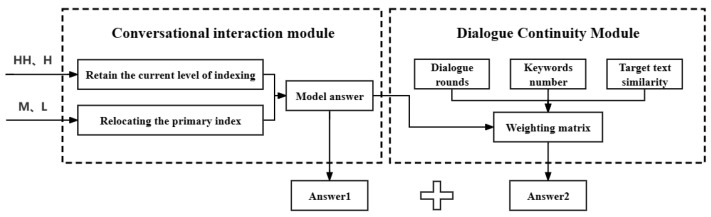
Sustainable dialogue system flowchart.

In this section, a sustainable dialogue interaction is carried out. In the proposed model, a secondary index is established to locate the specific dialogue and reduce the search range of the model, and the transformer model is used for question-answering interactions. From the second round of dialogue, according to the calculation rules of the degree of interest established in Section 4, the algorithm calculates the user input sentence’s level of interest and assesses whether the topic remains within the current domain or indicates a shift, thereby defining the scope of the primary-level index. Subsequently, the second-level index is carried out, and the answer is output using the model. Then, according to the number of dialogue rounds and the keywords of each text in the dialogue corpus, the normalized degree of interest of the dialogue text is assigned. In the first-level indexing result, the text with the highest value is identified by calculating the similarity and the text’s degree of interest. The flowchart of this section is shown in Figure 4.

### 5.1. Topic Transition

After the interest level calculation in Section 4, the user input statement will be assigned an interest level rating. If the user has a high or medium level of interest in the current topic, the user will continue with the current topic, and if the user has a fair or low level of interest in the current topic, the user will change the topic. In this section, the rules and methods of topic transition are described in detail. In the first round of a conversation, the parameter t is set to record the first-level index sequence number. Starting from the second round of conversation, according to the rules of the calculation of interest, the interest level of the user input statement is calculated. If $Interest_{Topic}$ is HH or *H*, then the first-level index range is not changed, and the current topic will continue to be discussed; if $Interest_{Topic}$ is *M* or *L*, then the first-level index range is relocated, and the content of the current index range is excluded during the localization.

### 5.2. Question and Answer Model

In this section, the question and answer model used in the proposed model is described in detail. The sequence length of the input samples is set to *L* before input, and a coding operation is performed on the input data samples. First, all of the words are organized into word lists with the minimum level of granularity for the word vectors of individual words. The number of word lists is *N* after the organization is completed. Each word in the word list is then converted into a unique hot code, so that an L×N training sample matrix is obtained. The word embedding matrix $M_{we}$ is learned and the segment-embedding encoding $M_{se}$ is added; in this context, the word-embedding matrix is characterized by a dimensionality denoted as *D*, with the matrix size specified as N×D, and the segment-embedding encoding matrix is a T×D-sized matrix of *T* *D*-dimensional row vector collocation. The addition of positional coding information to the word vector information is incorporated into the positional information via linear transformation of the trigonometric function, which produces a temporal sequence with periodic variations, and the information is finally encoded to obtain the matrix $M_{pe}$. The final matrix after adding the positional information and the paragraph vocabulary information is obtained as follows:

(2) $$M_{e}=M_{we}+M_{se}+M_{pe}$$

Multiply with the three weight matrices of the attention mechanism [29]; respectively calculate the Query matrix $Q_{m}$, Key matrix $K_{m}$, and Value matrix $V_{m}$. The specific formula for the attention mechanism is as follows:

(3) $$Att\left(Q_{m},K_{m},V_{m}\right)=softmax\left(\frac{Q_{m}K_{m}^{T}}{\sqrt{d_{K}}}\right)V_{m}$$

where $d_{K}$ denotes the dimension of the Me matrix, the output of the attention computation is obtained according to the above formula, the attention features corresponding to the word vectors are spliced, and the linear projection is computed to yield the feature matrix *A*. From this, the residual linkage is used to merge $M_{e}$ with the obtained attention feature matrix *A*, and then the standard normalization is performed to yield $M_{a}$. $M_{a}$ is activated by the activation function ReLU to obtain the hidden layer matrix, and the hidden layer matrix is summed with Ma and normalized to yield the new embedding matrix ${M_{e}}^{\prime }$. The new embedding matrix ${M_{e}}^{\prime }$ is obtained by adding the hidden layer matrix with $M_{a}$ after normalization.

${M_{e}}^{\prime }$ is computed through multiple transformer layers to produce the matrix $M_{te}$ of size D×T. The last decoder computation produces the output. The new embedded matrix ${M_{e}}^{\prime }$ generated by the transformer decoder is used to calculate the output, which is then multiplied by the word-embedding matrix *W* to obtain the attention scores for each word corresponding to the word-embedding matrix, resulting in the output $M_{te}$. The attention score corresponding to each word in the word list is obtained via matrix multiplication. Finally, the softmax function is employed to compute the probability for each word in the output. The word with the highest probability is selected, and each word is recursively output to obtain the final complete sequence, which outputs the answer to question $A_{1}$.

### 5.3. Continuing Dialogue

In order to make the AI chatbot more human-like, this section proposes a method that can allow the conversation to continue after the initial question is answered. The number of rounds of conversation, the number of keywords, and the target text similarity matrix of each text in the conversation dataset are constructed; the matrix is normalized, the weights are assigned, and the final text score is calculated; and finally, the text content with the highest similarity to the text with the highest scores is calculated for the continuation of the conversation.

Statistical calculations are performed on the dialogue dataset, and the number of rounds of dialogue, the number of keywords, and the similarity with the target question of each dialogue dataset are constructed into a dialogue eigenvalue matrix F, where $r_{j}$ is the number of rounds of dialogue, $k_{j}$ is the number of keywords, and $a_{j}$ is the similarity with the target question:

(4) $$F=\left(\begin{array}{cccccc} r_{1} & r_{2} & \ldots & r_{j} & \ldots & r_{n}\\ k_{1} & k_{2} & \ldots & k_{j} & \ldots & k_{n}\\ a_{1} & a_{2} & \ldots & a_{j} & \ldots & a_{n} \end{array}\right)$$

Since the units of each eigenquantity in the dialogue eigenvalue matrix are different and cannot be calculated directly, the matrix *F* is normalized so that the normalized matrix is $F^{\prime }$, the elements of the normalized matrix are ${f_{ij}}^{\prime }$, and the elements of the matrix before normalization are $f_{ij}$. The formula is as follows:

(5) $${f_{ij}}^{\prime }=\frac{f_{ij}}{\sum_{j=1}^{n}f_{ij}}$$

Since each feature plays a role of different magnitudes in influencing the final result, the weights of the features in the matrix are calculated next, and the coefficient of variation is used to calculate the weights in this section. For each indicator, its mean and standard deviation are calculated. The mean value can indicate the typical level of the indicator, and the standard deviation indicates the degree of fluctuation in the indicator value. The coefficient of variation is calculated for each indicator. The smaller the coefficient of variation, the lower the volatility of the indicator value, and vice versa. According to the size of the coefficient of variation, weights are assigned to each indicator. Indicators with smaller coefficients of variation may be assigned larger weights because their values are relatively stable and more reliable, while indicators with larger coefficients of variation may be assigned smaller weights because their values are more volatile and less stable. The values associated with each indicator are multiplied by their respective weights, then weighted and summed to derive the composite indicator score. The formula for calculation is as stated below, where $V_{i}$ is the coefficient of variation of the *i*th feature and $w_{i}$ is the weight corresponding to the *i*th feature:

(6) $$V_{i}=\frac{\sqrt{\frac{1}{n}\Sigma_{j=1}^{n}\left({f_{ij}}^{\prime }-\frac{1}{n}\Sigma_{j=1}^{n}{f_{ij}}^{\prime }\right)}}{\frac{1}{n}\sum_{j=1}^{n}{f_{ij}}^{\prime }}$$

(7) $$w_{i}=\frac{V_{i}}{\sum_{i=1}^{n}V_{i}}$$

After calculating the weight of each feature, in the secondary indexed content, the score is calculated by the feature weights to locate the text with the highest score. The formula is as follows:

(8) $$Score_{j}=\sum_{i=1}^{n}w_{i}{f_{ij}}^{\prime }$$

Within this text, the content of the next text is calculated based on the highest-rated statement. This content is linked to the output of the dialogue interaction module to generate the full response. The details of Algorithm 1 calculating the feature score are shown below.
**Algorithm 1** Calculating the Feature Score Algorithm.**Require:** Dialog dataset vector set dialogue, target problem vector question, Number of rounds of dialogue $r_{i}$, Number of keywords $k_{i}$ **Ensure:** Conversation feature scoring Score
1:n=|dialogue|2:a,r,k,V=[]3:**for** i←1 to *n* **do**           ▹ Calculate the similarity of each group of dialogues in the dialogue dataset to the target question4:      $a_{i}=1-\frac{dialogue_{i}\cdot question}{\sqrt{dialogue_{i}\cdot dialogue_{i}}\cdot \sqrt{question\cdot question}}$5:**end for**6:**for** i←1 to *n* **do**                                          ▹ Feature normalization7:      $a_{i}^{\prime }=\frac{a_{i}}{\sum_{i=1}^{n}a_{i}},\;r_{i}^{\prime }=\frac{r_{i}}{\sum_{i=1}^{n}r_{i}},\;k_{i}^{\prime }=\frac{k_{i}}{\sum_{i=1}^{n}k_{i}}$8:      $a\leftarrow a_{i}^{\prime },r\leftarrow r_{i}^{\prime },k\leftarrow k_{i}^{\prime }$9:**end for**10:$feature=[a_{i},r_{i},k_{i}]$11:**for** i←1 to *n* **do**                            ▹ Calculate the coefficient of variation for each feature12:      **for** *f* in feature **do**13:            $V_{i}=\sqrt{\frac{1}{n}\sum_{i=1}^{n}\left(f_{i}-\frac{1}{n}\sum_{i=1}^{n}f_{i}\right)}/\left(\frac{1}{n}\sum_{i=1}^{n}f_{i}\right)$14:            $V\leftarrow V_{i}$15:      **end for**16:**end for**17:S=sum(V)                                    ▹ Summation of coefficients of variation18:**for** *v* in *V* **do**19:      **for** i←1 to *n* **do**20:            $Score=v/S\cdot (a_{i}+r_{i}+k_{i})$21:      **end for**22:**end for**23:**return** Score


## 6. Experiments and Results

The CPU used in the experiment is an Intel Core i7-8550U, the main frequency is 1.8 GHz, and the software is PyCharm Community Edition 2020. The programming environment is python3.7.

### 6.1. Dataset

In our experiment, we chose the CMU_DoG dataset [27], which is a public dataset of document-based text conversations. See Section 3.2 for details. This dataset comprises designated documents focusing on Wikipedia articles discussing popular movies. It encompasses a total of 4112 conversations, averaging approximately 21.43 sessions per conversation.

### 6.2. Model and Evaluation Criteria

Following the experiment, the Icc_dialogue model underwent testing, revealing its practicality in emotional semantic perception, and we conducted comparative experiments with the EmpDG model and the DDMN model [30,31].

Evaluation criteria are very important in the design of dialogue systems. Reasonable and correct evaluation criteria can adequately assess the development of technology and lead dialogue systems in a better direction. Unlike task-based dialogue systems, which are evaluated based on the completion of a specific domain task, evaluating open-domain dialogue systems poses a challenge due to the inherently open-ended nature of such dialogues, as they lack fixed standard responses. Currently, open-domain dialogue systems are primarily assessed using two evaluation approaches: automated evaluation and manual evaluation [25,32,33]. Manual evaluation can be used to evaluate the answer sentences generated by the model from the perspective of real answer sentences, while automated evaluation is a widely used machine evaluation algorithm that takes into account the evaluation speed, so that objective evaluation can be carried out on each sentence generated by the model. This section will evaluate the algorithm from both of these aspects.

### 6.3. Automatic Evaluation

In terms of automatic rating, we used average response length, emotional consistency, and BLEU [25]. Long reply statements usually show that the user cares about the conversation and is willing to discuss the topic in depth, which can make the other person more willing to continue the dialogue and establish a deeper communication. We thus chose the average response length as an automatic evaluation indicator. Our research involves sentiment calculation, and the contextual consistency of emotions will result in better communication experiences for users, so the consistency of emotions can also be used as one of our references. BLEU measures the output result by comparing the similarity between the model output answer and the standard answer, and is mainly used to measure the accuracy.

Long responses can contain more information, ideas, and details, thus enriching the conversation, providing more valuable information, and prompting the other person to be more engaged in the discussion. Long response statements often help build more interesting and meaningful conversations. Therefore, we calculated the average reply statement length. The length of each reply under each model is counted and the average reply length is calculated in units of words, as shown in Table 3.

From the statistical results of the average reply statement length of each model based on the CMU_DoG dataset, it can be seen that the average reply statement length of the Icc_dialogue model is higher than that of other models. A higher average reply length allows the model to deepen users’ interest in the current dialogue, and at the same time, the longer reply statements can provide more valuable information, which prompts the users to be more engaged and participatory.

**Figure 5:**
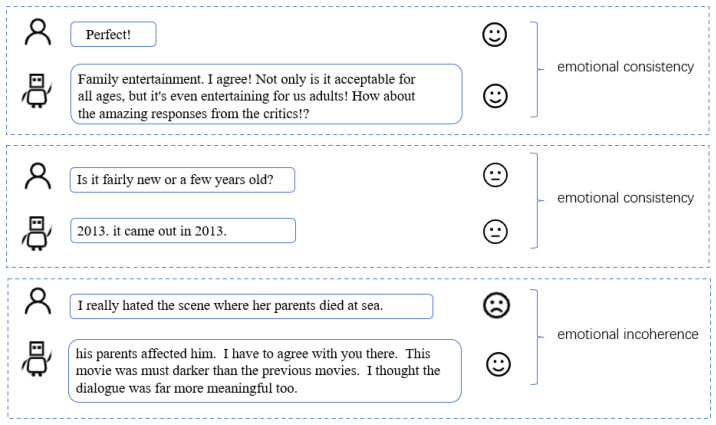
Affective congruence schema.

Because this article aims to compute the degree of interest of emotional words to determine interest and topic transition, assessment of emotional consistency in added to the experiment. An experiment using the SentimentIntensityAnalyzer class for sentiment analysis is used, in which the statement is entered into the tool to obtain a calculated sentiment score. Positive emotions are reflected by total scores surpassing 0, whereas scores falling below 0 indicate the presence of negative emotions and a score close to 0 means neutral. If the scores are in the same range, it indicates an agreed-upon mood, and if they are not, it indicates a disagreement. The emotional consistency of the dialogue context is calculated based on this level of agreement, as shown in Figure 5 below.

Through the dialogue calculation of the test set, the emotional consistency of the model’s response results was shown to reach 83.6%, indicating that our method is feasible and maintains a general emotional consistency.

BLEU (bilingual evaluation understudy) [25] was originally used in the field of machine translation to evaluate translation results by comparing the degree of similarity between a systematic translation constructed by an expert and multiple computer-generated responses. BLEU is mainly used to measure precision, i.e., the accuracy of a translation or a response. The similarity ranges from 0 to 1, with a higher score approaching 1 indicating superior translation quality. In the question and answer field, such as in the case of dialogue systems, this index is usually used. The overall formula for BLEU calculation is shown below:

(9) $$\mathrm{BLEU}=\mathrm{BP}\times \exp\left(\sum_{n=1}^{N}W_{p}\times {\mathrm{logP}}_{n}\right)$$

(10) $$\left.\mathrm{BP}=\left\{\begin{array}{cc} 1 & lc>lr\\ \exp\left(1-\mathrm{lr}/\mathrm{lc}\right) & lc<lr \end{array}\right.\right.$$

where lc is the length of the answer and lr is the length of the shortest reference answer. BLEU calculates the accuracy rate of the answers 1-gram, 2-gram, …, N-gram, where usually the value of N is set to 4. Here, Pn refers to the accuracy rate of N-gram, Wn denotes the weighting factor for N-gram (which is usually set to a uniform weight), and BP serves as the penalization factor, falling below 1 when the length of the answer sentence is shorter than the shortest reference sentence. The 1-gram accuracy rate of BLEU represents the degree of similarity with the original answer sentence, with the fluency of the generated answer being indicated by the remaining n-grams. The Natural Language Toolkit (NLTK) [34] is a Python library for processing human language data. It provides easy-to-use interfaces and resources for processing text data, performing language analysis, and building natural language processing (NLP) applications. In this thesis, NLTK is used to calculate BLEU. The statistical results of the BLEU indicators for each model are shown in Table 4.

**Figure 6:**
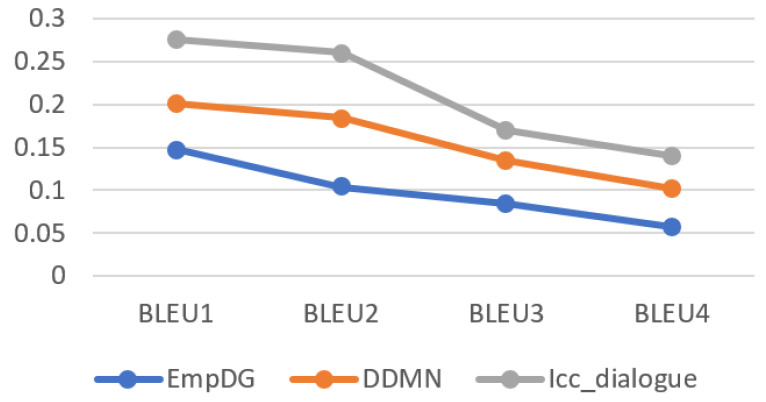
Line chart of statistical BLEU results of each model.

Line charts of the statistical results of the BLEU indicators for each model can be seen in Figure 6. The accuracy rate of the reply sentences generated by the Icc_dialogue model proposed in this paper is significantly higher than all the other models used for comparison, which indicates that the proposed model generates statements with higher similarity to the real replies.

Although BLEU is a widely used evaluation metric, it has some limitations. For example, it focuses mainly on n-gram matching and ignores higher-level factors such as syntactic structure and semantic meaning. Evaluations of dialogue models are more complex because they need to take into account more semantic and contextual information than just the surface matching of texts. Open-domain dialogues are inherently open-ended, with no fixed standard response. Therefore, the statistical scores of the BLEU metrics are not very high and can only be used as a basis for model evaluation. We therefore chose to perform a manual evaluation.

### 6.4. Manual Evaluation

In this paper’s manual validation method, 20 sets of question-and-answer dialogues were randomly selected to form a test set for manual assessment, and 20 validators were employed. In this paper, the manual assessment method adheres to the evaluation metrics commonly employed in single-round assessments. The manual assessment metrics and their explanations are as follows: (1) Appropriateness: Evaluates whether the answer sentences generated in the generative model based on multi-round dialogues are logical and whether the generated answer sentences are suitably matched to the current answer context. (2) Informativeness: Evaluates whether the answer sentences generated by the multi-round dialogue-based generative model are informative and whether the generated answers are related to the context of the user’s question. (3) Grammaticality: Evaluates whether the answer sentences generated by the multi-round dialogue generative model are fluent, whether the fluency is in accordance with the logic of normal dialogue, and whether the generated answer sentences are grammatical or there are obvious grammatical errors that are contrary to real dialogue. The validator will score the responses to 20 questions answered by the different models in three aspects on a scale of 0–10, where 0 means very noncompliant and 10 means very compliant. Table 5 indicates the performance of the different models that have been manually evaluated on the CMU_DoG dataset.

**Figure 7:**
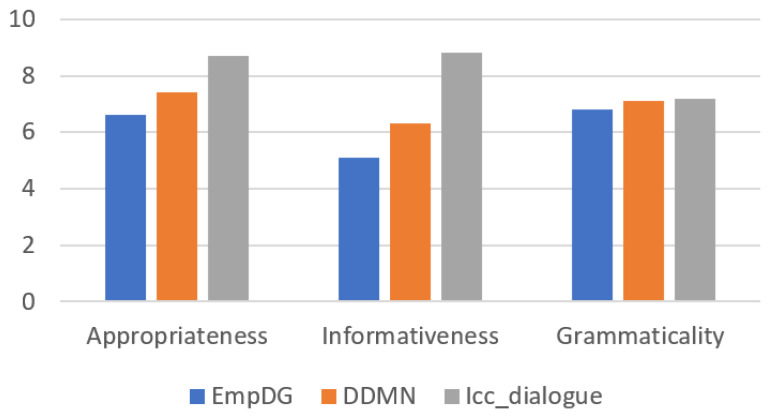
Manual evaluation performance histogram of each model.

In terms of evaluation metrics for human assessment, human assessment focuses more on the scope that is not covered by machine metrics, and thus can be used as an evaluation metric from another perspective. Figure 7 illustrates that the Icc_dialogue model, introduced in this paper, outperforms in terms of appropriateness, informativeness, and grammaticality. This indicates that after adding the calculation of interest and continuous dialogue, the dialogue model is more in line with real-life language habits and further improves the Q&A form of the traditional dialogue model.

The following figures shows a comparison between this model and ChatGPT, which is currently a highly popular conversation model.

**Figure 8:**
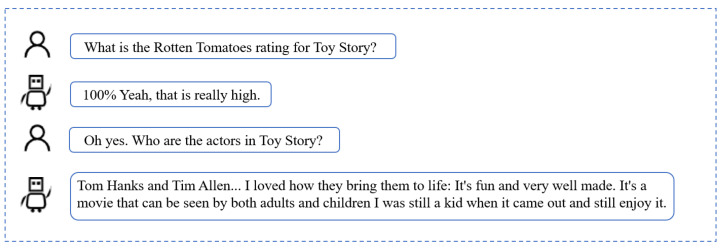
Example dialogue from the Icc_dialogue model.

**Figure 9:**
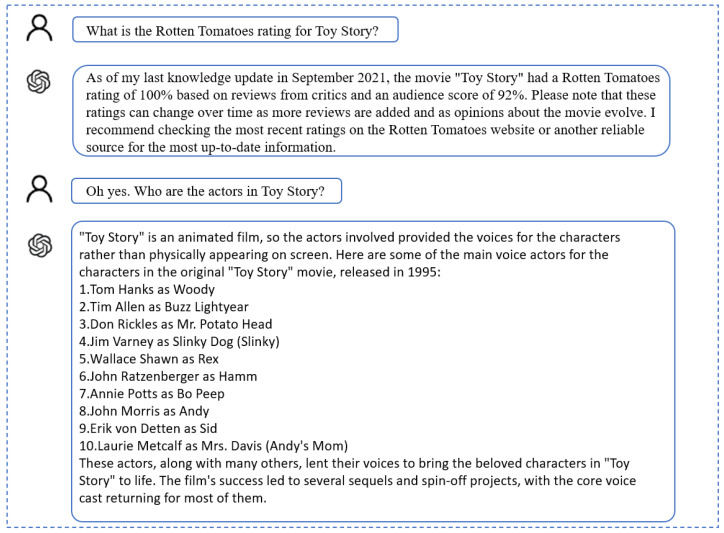
Example dialogue from ChatGPT-3.5.

As seen in Figure 8 and Figure 9, although ChatGPT’s answers are more detailed and comprehensive, its use is more like a search engine for the solution of the user’s problem. Meanwhile, the Icc_dialogue model proposed in this paper is more similar to everyday conversation and meets the needs of the user’s daily conversations.

## 7. Discussion

The experimental findings indicate the high reliability of incorporating interest level calculations and continuous dialogue into the conversation. The introduction of sentiment calculation and user interest calculation allows our model to understand and respond to user emotion more intelligently, thus improving the naturalness and quality of the dialogue. We also introduced the topic-switching mechanism, which dynamically adjusts the direction of the conversation according to the level of interest. By assessing the level of interest after the model’s answer, the model is able to flexibly decide whether to switch topics, thereby keeping the user’s interest in the conversation high. This innovation makes the dialogue more engaging and interactive.

In addition, in terms of continuing the conversation after answering the initial question, we adopted a strategy based on the number of dialogue rounds, the number of keywords, and the target text’s similarity matrix. By taking these metrics into account, we were able to construct a model that can determine the best route for continuing a conversation. This helps solve the problem of current models not actively continuing the dialogue after answering the initial question, and our model thus avoids situations that can lead to conversation interruption.

In the experimental section, we conducted both automatic and manual evaluations to verify the significant improvement of the Icc_dialogue model in terms of dialogue quality and user experience. The results of the automatic evaluation show that our model achieves higher performance in language-generation tasks. Through manual evaluation, we further confirm the effectiveness and feasibility of the Icc_dialogue model in practical applications.

In summary, the Icc_dialogue model proposed in this study has achieved positive results in improving the performance of the dialogue system. However, the method also has certain limitations. Currently, most robot hardware has limited computing power that cannot directly deploy the model in its entirety, so it needs to be deployed on a cloud server for invocation; however, cloud servers are subject to problems such as voice latency. In addition, the accuracy of special syllable recognition is another one of our model’s challenges.

## 8. Conclusions

This study integrated a voice interaction module, carried out emotion calculation based on the model’s generated answer, and formulated the calculation rules of user degree of interest. According to the calculation results of the degree of interest, the model is able to determine whether to switch topics in order to maintain the user’s significant interest. At the same time, the model in this study carries out topic continuation after the initial question is answered. Based on constructed dialogue rounds, keyword number, and the target text similarity matrix of each text in the dialogue dataset, the model normalizes the matrix, assigns weights, and calculates the final text score. In order to continue the dialogue with the user, the text content of the next sentence is based on text with the highest similarity to the text with the highest score.

Based on the analysis of the experimental results in this paper, it can be seen that incorporating the calculation of interest level and continuous dialogue into the conversation is meaningful. This experiment’s incorporation of emotional factors into human–computer dialogue makes the system more human-like, and also avoids the problem of the model not continuing the conversation after answering the initial question but instead waiting for the user to provide more information voluntarily, which leads to topic interruption when continuous dialogue is in progress.

Although this research already considers user emotion and topic continuation, emotions can be expressed in different ways, and the field of dialogue research has some limitations in this area. Our research group has conducted research on facial expressions and body language before, and has obtained some interesting results. In subsequent research, we will move towards the combination of these results with the research presented in this paper and strive to form a more perfect human–computer interaction system.

## Figures and Tables

**Table 1:** A summary of the current status of evaluation tools and open-domain and closed-domain human–computer dialogue systems.

	Research Status
**Evaluation tools**	Automatic evaluation metrics: BLEU is used to automatically evaluate machine translation metrics and is widely used in dialogue systems. ROUGE is primarily used to evaluate text summaries, but it also has applications in conversational systems. Dialogue quality index generation: Perplexity is used to measure the uncertainty of text generated by models. The lower the value, the better the model’s performance. Manual evaluation: User evaluation of a dialogue system’s performance is obtained through manual annotation or questionnaire survey.
**Open-domain** **human–computer** **dialogue systems**	Researchers have made progress in solving syntactic and semantic problems, highlighting efforts to improve robots’ anthropomorphic characteristics. Models such as Emotion Chat Machine (ECM) and Context-enhanced Neural Question Generation (CNQG) are introduced, which aim to consider affective consistency and improve interactivity in conversation generation, respectively. In addition, SentiGAN and C-SentiGAN models achieve automatic generation of text with different emotional labels through multiple generators and multi-class discriminators.
**Closed-domain** **human–computer** **dialogue systems**	Kang et al. [13] proposed a recommendation system based on conversational tasks, with experts gradually learning about user needs and recommending them products. Quan et al. [20] used the joint cepstrum distance method to identify emotions in speech, while Rao et al. [21] reviewed sentiment computing techniques based on semantic analysis, Kanjo et al. [22] used deep learning to classify multimodal sensor signals, and Chen et al. [24] used support vector machines and random forests as emotional classifiers.

**Table 2:** Topic interestingness calculation rules.

Topic Degree of Interest	Topic Interestingness Calculation Rules
$Interest_{Topic}=HH$	$Interest_{Top_{1}}=HH\cap Interest_{Top_{2}}=H,M$
	$Interest_{Top_{2}}=HH\cap Interest_{Top_{1}}=H,M$
	$\left(Interest_{Top_{1}}=L\cap Rate_{Interest_{Top_{1}}}<75\%\right)⋂Interest_{Top_{2}}=HH$
	$\left(Interest_{Top_{2}}=L\cap Rate_{Interest_{Top_{2}}}<75\%\right)⋂Interest_{Top_{1}}=HH$
$Interest_{Topic}=H$	$\left(Interest_{Top_{1}}=M\cap Rate_{Interest_{Top_{1}}}<65\%\right)⋂Interest_{Top_{2}}=H$
	$\left(Interest_{Top_{2}}=M\cap Rate_{Interest_{Top_{2}}}<65\%\right)⋂Interest_{Top_{1}}=H$
	$\left(Interest_{Top_{1}}=L\cap Rate_{Interest_{Top_{1}}}<50\%\right)⋂Interest_{Top_{2}}=H$
	$\left(Interest_{Top_{2}}=L\cap Rate_{Interest_{Top_{2}}}<50\%\right)⋂Interest_{Top_{1}}=H$
$Interest_{Topic}=M$	$\left(Interest_{Top_{1}}=M\cap Rate_{Interest_{Top_{1}}}>=65\%\right)⋂Interest_{Top_{2}}=H$
	$\left(Interest_{Top_{2}}=M\cap Rate_{Interest_{Top_{2}}}>=65\%\right)⋂Interest_{Top_{1}}=H$
	$\left(Interest_{Top_{1}}=L\cap Rate_{Interest_{Top_{1}}}<25\%\right)⋂Interest_{Top_{2}}=M$
	$\left(Interest_{Top_{2}}=L\cap Rate_{Interest_{Top_{2}}}<25\%\right)⋂Interest_{Top_{1}}=M$
$Interest_{Topic}=L$	$\left(Interest_{Top_{1}}=L\cap Rate_{Interest_{Top_{1}}}>=75\%\right)⋂Interest_{Top_{2}}=HH$
	$\left(Interest_{Top_{2}}=L\cap Rate_{Interest_{Top_{2}}}>=75\%\right)⋂Interest_{Top_{1}}=HH$
	$\left(Interest_{Top_{1}}=L\cap Rate_{Interest_{Top_{1}}}>=50\%\right)⋂Interest_{Top_{2}}=H$
	$\left(Interest_{Top_{2}}=L\cap Rate_{Interest_{Top_{2}}}>=50\%\right)⋂Interest_{Top_{1}}=H$
	$\left(Interest_{Top_{1}}=L\cap Rate_{Interestrop_{1}}>=25\%\right)⋂Interest_{Top_{2}}=M$
	$\left(Interest_{Top_{2}}=L\cap Rate_{Interest_{Top_{2}}}>=25\%\right)⋂Interest_{Top_{1}}=M$

**Table 3:** Average reply statement lengths of different models.

Model	EmpDG	DDMN	Icc_Dialogue
Sentence length (words)	8.29	11.67	13.19

**Table 4:** Statistical results of BLEU indicators for each model.

Model	BLEU1	BLEU2	BLEU3	BLEU4
EmpDG	0.148	0.104	0.084	0.057
DDMN	0.201	0.184	0.134	0.102
Icc_dialogue	0.276	0.260	0.170	0.140

**Table 5:** Manual evaluation of the performance of each model.

Model	Appropriateness	Informativeness	Grammaticality
EmpDG	6.6	5.1	6.8
DDMN	7.4	6.3	7.1
Icc_dialogue	8.7	8.8	7.2

## Data Availability

The datasets analyzed in this study are publicly available. The Sentiment140 dataset can be found here: http://help.sentiment140.com/for-students (accessed on 16 May 2009). The CMU_DoG dataset can be found here: https://github.com/festvox/datasets-CMU_DoG (accessed on 21 September 2018).
